# Levels of N-terminal pro-brain natriuretic peptide in brain injury patients

**DOI:** 10.1186/cc14538

**Published:** 2015-03-16

**Authors:** L Tsentsiper, E Kondratyeva, S Kondratyev, N Dryagina

**Affiliations:** 1 Russian Polenov's Neurosurgical Institute, Saint-Petersburg, Russia

## Introduction

Currently, the most common way to predict the outcome of acute brain damage is to study the level of protein S-100 in the serum. This method lacks specificity as the concentration of protein S-100 significantly increases with age, more for men than women, and there are no data on prognostically significant changes in the level of S-100 after removal of the tumor and cerebral hemorrhages. Endothelins, vasopressin, some cytokines, excess sodium or calcium in serum, activation of the sympathoadrenal system, and tachycardia are the stimulants of brain natriuretic peptide production. The rise of the natriuretic peptide level in cases of acute brain damage has a functionally adaptive nature, based on vasodilation, diuretic action peptide and ability to reduce sympathoadrenal system activity. Thus, we can suppose that the more severe the damage, the higher the stimulation of natriuretic peptide. In this study we investigate the level of N-terminal pro-brain natriuretic peptide (NT-proBNP) in patients with severe brain damage and find correlation between the level of peptide and outcome.

## Methods

We studied 110 patients having brain injuries of various origins. All patients were divided into four groups. All patients were 20 to 72 years old, 58 men and 52 women. Group 1 (*n *= 17) - acute TBI, group 2 (*n *= 29) - patients operated on for the brain tumor, group 3 (*n *= 36) - hemorrhagic stroke, group 4 (*n *= 28) - vegetative state. We measured the level of brain natriuretic peptide on days 1 to 3, and then every 7 days for 21 days.

## Results

All patients with severe acute brain damage (groups 1, 2, 3) had a level of NT-proBNP higher than normal (normal 0 to 125 pg/ml). Significant difference in values between the groups was not observed. Level of NT-proBNP above 700 pg/ml and/or the absence of its reduction to normal dynamic indicators was marked by an unfavorable outcome of the disease - severe disability (*n *= 25) or death (*n *= 18). For patients from group 4 regardless of their age, sex, severity of condition and treatment results in a level of NT-proBNP below 250 pg/ml.

## Conclusion

In cases of acute severe brain damage the level of NTproBNP significantly increased. Correlation between the level of NT-proBNP and etiology of acute brain damage was not observed. If the level of NT-proBNP is above 700 pg/ml and/or in the absence of its reduction to normal, then poor outcome of the disease - severe disability or death - can be predicted. Level of NT-proBNP cannot be used as an indicator for the severity of the status for patients in a vegetative state in contrast to patients with acute brain damage.

